# A Model Practice Act

**Published:** 1914-07

**Authors:** H. Sheridan Baketel

**Affiliations:** New York, Editor Medical Times


### A Model Practice Act.

BY H. SHERIDAN BAKETEL, A. M., M. D., NEW YORK, EDITOR MEDICAL TIMES.

The present medical registration laws in the United States are imperfect in many details, in some cases in their basic principles. In view of this well recognized fact, and seeking a solution of the vexatious problem, I asked assistance of the presidents of several State medical examining boards last spring, having in mind the enactment of a national medical registration law patterned after the Conjoint Board of the Royal Colleges of Physicians and Surgeons of Great Britain. By meeting the requirements of such a body, the successful candidate would be enable to practice his profession in any part of the United States or its possessions.

This symposium, published in the *Medical Times* of June, 1913, showed the impossibility of such a law on account of our constitutional belief in States' rights. Former Assistant District Attorney Almuth C. Vandiver, counsel to the Medical Society of the County of New York, and one of the best informed legal authorities on medical legislation in America, pointed out that "Without an amendment to the Constitution of the United States enlarging the powers of Congress to that extent, no medical registration act providing for the qualifications of medical practitioners and registration of their licenses to practice medicine with the proper officials, is possible in this country."

It is necessary, therefore, to seek another solution and that is found in the adoption by the various Commonwealths of a uniform practice act, based on an equalization of standards which will make reciprocity between each of the several States a reality.

In this way alone can we obtain national reciprocity and mete out full justice to the physician who may desire to change his location. A uniform practice act can best be formulated through the good offices of the Federation of State Medical Boards of the United States. If this body can agree upon a model law, we can undoubtedly depend upon the various State associations to place it upon the statute books of the individual States.

In the first place we should determine whether the model practice act should be definitive or restrictive. Practically all of our State laws today are of the second class. They define the practice of medicine and prohibit all unqualified and unlicensed persons, with certain specific exceptions, from practicing what is defined to be medicine.

Those favoring the definitive statute advocate proper education to the end that the laity will understand that physicians must possess certain qualifications and that they may also know what qualifications may be expected of such persons as optometrists, chiropodists, osteopaths, Christian Scientists et al.

Advocates of a restrictive statute do not believe the time is ripe for widespread education of the public and feel that for the proper protection of the general health of the community the practice of medicine should be restricted to those properly qualified and duly licensed.

I would advocate the creation in each Commonwealth of a State Board of Medical Examiners, composed of six doctors of medicine, appointed by the Governor *only* upon the recommendation of the State medical societies. This would prevent the appointment of political henchmen—a misfortune to which some States are at this time subjected. These men should be appointed in pairs, their terms expiring in two, four and six years, respectively.

The educational requirements for practice should be clearly defined and uniform, so far as our governmental and legislative system will permit. It seems absurd that imaginary State boundary lines should preclude properly licensed practitioners from pursuing their profession in regions other than those in which they are licensed. They are now excluded upon the theory that the educational standards and legal requirements are dissimilar.

Under this uniform act it should be specified that each applicant should have had at least one year of collegiate training equal to the work of the freshman year in a standard college, including physics, chemistry and biology (both didactic and laboratory work), elementary physiology and French or German.

He should be graduated from a medical college, recognized by the American Association of Medical Colleges as being a standard institution in every particular, and he should in addition have had at least one year as an interne in a hospital or in post-graduate clinical study in a recognized post-graduate medical school. After a rigid investigation of the candidate's credentials (and in this particular some boards are lax) he should be admitted to an examination in the practice of medicine and diagnosis, bacteriology and pathology, general surgery and its various special branches, materia medica and therapeutics, obstetrics and gynecology, and hygiene and preventive medicine.

Anatomy, physiology and chemistry are fundamentals belonging solely in the medical school. If the candidate is not thoroughly grounded in those branches his ignorance will make itself manifest in the examiantions in the professional subjects and he will fail to qualify in consequence. It is incongruous to examine so religiously prospective practitioners in subjects passed in the first and second years of their medical education and omit therapeutics and materia medica, one of the most essential subjects in medicine.

The examination should be written, oral and clinical. The brightest theoretical student may be absolutely unable to apply his knowledge practically. In no other way than an oral, clinical examination can a man's skill as a physician be accurately gauged.

In advocating practical examinations, Dr. Otto V. Huffman, Secretary of the New York Board of Medical Examiners, says in the *Medical Times,* June, 1913:

"There are two other reasons why it should be a practical examination. The first is the reflex effect of causing the medical schools to give proper practical training and the student to seek experience outside of the medical school curriculum. The other is the very desirable effect of practical training in medicine on moral development. There is no other way by which the moral tone of the profession could be raised more surely and more quickly. The student would take a true delight in preparing for a thorough practical examination given in a leisurely manner; not simply looking into a microscope and stating what is seen, looking over one or two old chronic cases, or designating the use of a few instruments. The wholesome effect of proper thoroughly practical examinations on both the teacher and pupil can hardly be estimated. Under such a plan it would be a pleasure to teach and train students. It would then become a pleasure to study medicine and to prepare for the test."

These examinations could be given in the free hospitals and dispensaries, where there is an abundance of clinical material. By means of this thorough insight into the candidate's professional knowledge, every newly qualified physician would be a safe person to entrust with the lives of those given into his keeping. This cannot be said of some of the callow, illy prepared, young medical graduates, who are turned loose upon many communities today.

The fee for this medical examination should be $50—on account of the added time the conduct of the practical examinations would take. Under our present plan the examinations are held under the watchful eyes of assistants and the examiner prepares the questions and reads the answer thereto in his own office. With a practical examination he would of necessity be compelled to give two or three days' time at each examination period for the oral and clinical work.

The license to practice once granted, it should be held by the recipient only during good behavior.

The act should contain a clear and definite statement of the method of procedure for the revocation of the license issued to a physician. The accused should be given a public hearing by the board, with privilege of counsel, and the findings of the board should be subject to review by the highest court of the State. The license should be suspended for stated periods for such offenses as advertising to cure chronic diseases, venereal diseases or female irregularities and for unprofessional conduct. It should be revoked permanently upon conviction of a felony, or conviction of the crime of abortion or the selling of articles to produce abortion, or the unlawful sale of habit-forming drugs. The license should also be annuled upon conviction of any fraud and deceit in practice and what constitutes deceit and fraud and unprofessional conduct should be included in the act.

Provision should also be made for the revocation of the license if any physician is judicially declared to be insane, to be a dipsomaniac or to be a drug habitué. I would include advertising as before mentioned as a cause of revocation, had not the Supreme Court of Colorado recently declared such a law unconstitutional.

If the practice act is definitive, there need be no statutory exemptions.

If it be restrictive, the exemptions should be as few as possible.

The question of the practice of the religious tenets of a church, such as Christian Science, new thought, and the like, should be completely disposed of by a concise statement of the limits within which the individual must remain for the purpose of protecting himself, even against his own wishes, and protecting the State.

A statutory statement of the effect of a pardon issued to any physician after the conviction of a crime, either by the President of the United States or the Governor of a State, should be contained in the act.

The candidate's examination papers should be kept in the office of the board of medical examiners located at the State Capitol with a clerk provided by the State to attend to all matters in connection with the preliminary work of answering questions regarding examinations and to be responsible for all papers.

The name of every licentiate in medicine should be kept in the office of the State Board of Medical Examiners. When the successful candidate registers his license with the county clerk, register or other county official, that the officer should be directed by law to immediately send the facts in the case, name, address, medical school of graduation and date of registration of license to the State Board of Medical Examiners, so that the board might have in its possession at all times the pertinent facts connected with every practitioner of medicine in the Commonwealth. The penalty for unlicensed practice should not be overlooked.

For working out a model practice act, I have touched lightly upon some features in license and registration, revocation of license, exemption from operation of statute and administration which should in the preparation of the act be clearly defined, for unless every contingency is fully covered by being prescribed by statute trouble will result.

Medical unity should exist in every State, and the proportion of regular, homeopathic and eclectic members of the board of medical examiners should be in proper proportion to the number of licensed practitioners of those schools in the State.

If a practice act, based on lines as set forth herein, can be placed upon the statute books of the several States, we shall be enabled by the regulation and equalization of standards to enjoy all the benefits of national reciprocity.—*The Centaur.*

---

The charter of the New Orleans Post-Graduate School of Medicine was filed on Thursday, June 11th. The organization is composed of prominent members of the medical profession of New Orleans not connected with Tulane University, and its object is to offer to graduates in medicine a post-graduate course in medicine, surgery, and the specialties. The first board of directors is composed of Dr. Homer Dupuy, President; Dr. William Kohlmann, Vice-President; Dr. Joseph A. Danna, Secretary; Dr. Oscar Dowling, Dr. O. L. Pothier, Dr. Abe Nelken, Dr. J. M. Elliot, Dr. C. G. Cole, Dr. T. J. Dimitry.

